# Artificial intelligence interaction is not equivalent to social interaction: Rethinking artificial intelligence‐mediated digital exposure in early childhood

**DOI:** 10.1002/ped4.70086

**Published:** 2026-09-12

**Authors:** Ayhan Çeri, Hüseyin Yılmaz

**Affiliations:** ^1^ Department of Pediatrics Dr. Ali Kemal Belviranlı Maternity and Children's Hospital Selçuklu Konya Turkey; ^2^ Pediatric Cardiology Unit Konya City Training and Research Hospital Karatay Konya Turkey

**Keywords:** Artificial intelligence, Children, Cognitive development, Digital media, Interaction, Pediatric, Socio‐emotional

## Abstract

The increasing integration of artificial intelligence (AI) into children's daily environments challenges traditional approaches to evaluating digital exposure. Although existing research has primarily focused on screen time, AI‐based interactions introduce qualitatively different dynamics that remain insufficiently conceptualized. This narrative review aims to synthesize current evidence on digital media use and emerging evidence on AI‐based interaction to propose a novel framework distinguishing AI engagement from traditional screen exposure. Findings indicate that traditional screen exposure is associated with adverse outcomes in language development, attention regulation, and social interaction, although these associations are not uniform and are moderated by content and parental involvement. In contrast, AI‐based systems provide adaptive and personalized interactions that may enhance cognitive engagement, including learning and problem‐solving. However, these systems lack genuine emotional reciprocity and may create an illusion of social interaction. Based on these findings, a spectrum model of digital exposure is proposed, illustrating a trade‐off between increasing cognitive stimulation and decreasing opportunities for authentic social development across passive, interactive, and AI‐based media. These findings highlight the need to move beyond time‐based metrics toward frameworks that consider interaction quality, and updated clinical and policy approaches are required to address the developmental implications of AI‐integrated childhood environments.

## Graphical abstract

Artificial intelligence introduces a developmentally distinct form of digital exposure. As interaction complexity increases from passive media to AI‐based systems, cognitive engagement may increase, whereas opportunities for authentic human social interaction may decrease, highlighting the need for AI‐specific pediatric assessment frameworks.

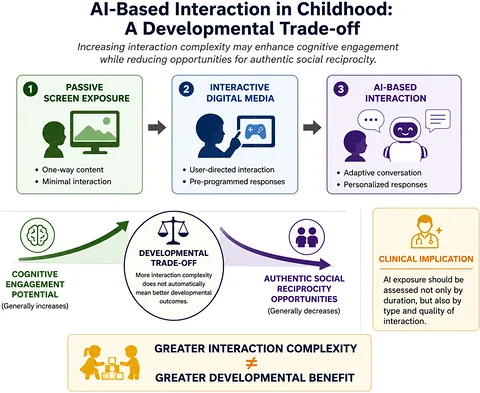

## INTRODUCTION

Over the past two decades, digital technologies have reshaped childhood from a predominantly physical and social experience into one increasingly mediated by screens and, more recently, artificial intelligence (AI).^1^, ^2^, ^3^ These changes have influenced pediatric guidelines and parental practices, with “screen time” becoming a central metric for evaluating digital exposure in early childhood.^4^ Recently, the digital environment has shifted in more fundamental ways. Unlike traditional media and conventional interactive applications, AI‐driven systems, including conversational agents, adaptive learning platforms, and personalized digital companions, introduce adaptive and responsive forms of interaction.^5^, ^6^ Rather than simply consuming content, children increasingly engage in dynamic exchanges with systems that simulate aspects of human communication.^7^ Despite this shift, AI‐based engagement is still commonly conceptualized within the broader framework of screen time.^8^ This mismatch matters because time‐based metrics were designed to capture exposure to largely noninteractive content; they were not built to register whether a digital system responds contingently to a child, adapts to the child's behavior, or simulates a social partner, which are precisely the properties that distinguish AI‐based interaction. Consequently, two children with identical screen‐time totals could have qualitatively different digital experiences, one watching a fixed video and the other engaged in adaptive dialogue with a conversational agent, yet current frameworks would classify their exposure as equivalent. However, AI‐mediated interaction differs from passive screen exposure in important ways. Although traditional screen use is often characterized by passive consumption and reduced interaction, AI systems may increase cognitive engagement through personalization and real‐time feedback.^5^, ^9^ At the same time, they lack genuine emotional reciprocity and may simulate social connection without supporting authentic interpersonal development.^8^ What remains unknown, and what this review aims to address, is how these competing cognitive and social consequences of AI‐based interaction should be conceptualized and weighed against one another within a single framework of digital exposure, rather than assessed as isolated benefits or risks.

This conceptual ambiguity raises an important question: can AI‐mediated interaction be adequately understood using frameworks originally developed for traditional screen exposure? Recent work has begun to question the applicability of conventional “screen time” models to emerging AI‐based interactions.^8^ This conceptual argument is reinforced by a fuller empirical and theoretical treatment of AI‐chatbot cognitive impact,^8^, ^10^ so the present framework does not rest on a single short commentary alone. Building on this perspective, this review adopts a developmentally grounded approach focused on early childhood, examines diverse forms of AI interaction, and proposes a conceptual framework, the digital exposure spectrum, to explain how increasing interaction complexity may differentially influence cognitive engagement and social reciprocity. The review further considers the clinical and policy implications of these developmental shifts. This review is accordingly best understood as a hybrid narrative synthesis and conceptual proposal: It first synthesizes existing empirical evidence on screen exposure and emerging AI‐based interaction and, building on that evidence, then puts forward a new conceptual model rather than testing a pre‐specified hypothesis.

## METHODS

This study employed a narrative review approach to synthesize the literature on digital media use, AI‐mediated interaction, and child development. Relevant publications spanning mainly the period between 2010 and 2026 were identified through PubMed, Scopus, and Google Scholar, using combinations of search terms including “screen time,” “digital media,” “artificial intelligence,” “conversational agent,” “chatbot,” “social robot,” “early childhood,” and “child development.” A small number of seminal earlier sources, identified through reference‐list screening of the articles retrieved by the primary search, were added by hand search when they remained the most directly relevant citation available for a specific claim; this accounted for the small number of pre‐2010 references in the final list. Reference lists of relevant articles were also screened for additional sources, and the eligibility of candidate studies was assessed by the authors based on topical relevance to screen exposure, AI‐mediated interaction, or child development, prioritizing peer‐reviewed and developmentally focused sources over convenience matches.

Studies addressing the cognitive or socio‐emotional effects of screen exposure, interactive media use, and AI‐based interaction with developmental relevance were considered. AI‐based interaction has been operationalized to include generative AI tools, conversational agents and chatbots, adaptive learning platforms, and AI companions or social robots. For clarity, three additional terms used throughout this review are defined explicitly: “traditional media” refers to noninteractive content delivery such as television or video, where the child is a passive recipient; “conventional interactive applications” refers to pre‐programmed digital tools such as apps, games, and e‐books that respond to user input according to fixed, nonadaptive rules; and “interaction complexity” refers to the degree to which a digital system adapts its behavior contingently and in real time to a specific child's input, ranging from minimal (traditional media) to substantial (AI‐based systems). Throughout this review, “AI interaction,” “AI‐mediated interaction,” and “AI exposure” are used interchangeably to refer to a child's engagement with AI‐based systems as defined above; no distinct technical meaning is intended by the variation in phrasing. Given the limited empirical literature on AI use in early childhood (defined as birth through 5 years of age), conceptually relevant studies involving older children, adolescents, or related technologies were also included where informative; such studies are identified as such at first citation, and any extrapolation to early childhood is noted explicitly.

Priority was given to systematic reviews, meta‐analyses, and peer‐reviewed empirical studies, whereas selected policy reports and conceptual papers were used to provide theoretical and contextual grounding. Findings were synthesized conceptually to identify patterns across forms of digital interaction and to develop the proposed digital exposure spectrum framework.

Generative AI tools (GPT‐5.6 Sol, OpenAI) were used during manuscript preparation to assist with draft concept development, construction of the literature pool, language refinement, text organization, and editorial improvements. The authors reviewed, verified, and revised all AI‐assisted content and take full responsibility for the accuracy, interpretation, and conclusions presented in the manuscript. No AI tool was involved in study design, literature selection, data interpretation, or scientific decision‐making.

## TRADITIONAL SCREEN EXPOSURE AND CHILD DEVELOPMENT

### Cognitive outcomes

A large body of research has examined the relationship between screen exposure and cognitive development in early childhood, with particular emphasis on language acquisition and attentional processes. Several longitudinal and meta‐analytic studies have reported associations between increased screen time and delays in expressive language development, especially in children under 5 years of age.^1^, ^11^ In a meta‐analysis of 38 studies, greater quantity of screen use was associated with lower language scores (*r* = −0.14; 95% CI: −0.18 to −0.10).^1^ This dose–response relationship, including how screen time interacts with other behavioral factors to shape cognitive outcomes, has been examined in more detail in a recent scoping review focused on children and adolescents.^12^ These effects are often attributed to the displacement of interactive, language‐rich environments, such as caregiver–child communication, which are critical for early linguistic development.^13^, ^14^

In addition to language outcomes, screen exposure has been linked to alterations in attention regulation. High levels of early screen use have been associated with shorter attention spans, increased distractibility, and difficulties in sustained focus during later childhood.^15^, ^16^, ^17^ Rapidly changing visual stimuli and reward‐driven content, commonly found in digital media, may condition children to expect high levels of stimulation, potentially undermining the development of endogenous attention control mechanisms.^15^, ^17^ In a 13‐month longitudinal study, children exceeding the American Academy of Pediatrics’ recommended 2‐h daily media limit showed significantly greater subsequent attention problems than those below it.^16^ Neuroimaging evidence further suggests that early and intensive screen use may be associated with structural and functional differences in brain regions that support language and cognitive control.^18^, ^19^ These associations are not uniform across all types of media use, and the relationship between screen time and cognitive outcomes remains complex and context dependent.^20^, ^21^

### Social‐emotional effects

Beyond cognitive development, traditional screen exposure has significant implications for children's social and emotional development. One of the most consistently reported concerns is the potential for reduced social interaction. Increased screen time may displace opportunities for face‐to‐face engagement with caregivers and peers, which are essential for the development of empathy, emotional regulation, and social communication skills.^22^, ^23^ Moreover, excessive screen use has been associated with higher levels of social withdrawal and behavioral difficulties in some studies.^2^, ^24^ Children who spend more time engaged with screens may have fewer opportunities to practice interpreting social cues, such as facial expressions and tone of voice, which are fundamental components of socio‐emotional learning.^22^, ^25^ In one large population‐based study (mean screen time 3.20 h/d·), children and adolescents exceeding roughly 1h/d · of screen time showed progressively lower psychological well‐being, and high users (7+ h/d = ·) were twice as likely to have an anxiety or depression diagnosis compared with low users.^25^

Parental interaction plays a particularly crucial role in this context. When screen use occurs in isolation, its negative effects on social development may be amplified.^23^, ^26^ Conversely, the absence of shared attention and joint engagement during media use can reduce the potential for meaningful social learning.^3^, ^25^ Thus, the impact of screen exposure on socio‐emotional development is closely tied to the broader relational environment in which it occurs.

### Moderating factors: Content and parental involvement

Importantly, the effects of screen exposure are not solely determined by duration but are significantly moderated by content and context.^4^, ^13^, ^27^ High‐quality, age‐appropriate educational content has been shown to support certain aspects of cognitive development, particularly when it is designed to promote active engagement rather than passive consumption.^13^, ^14^ In contrast, fast‐paced or noneducational content may exacerbate attentional and behavioral challenges.^17^

Parental involvement represents another critical moderating factor. Co‐viewing and active mediation, where caregivers engage with children during media use, ask questions, and provide explanations, can transform screen time into a more interactive and developmentally supportive experience.^3^, ^28^ However, recent evidence also indicates that parents’ own technology use may inadvertently displace responsive caregiver–child interaction, even when children's screen time is limited.^28^, ^29^ Such practices can help children relate digital content to real‐world contexts, which may support learning and social connection.^4^, ^29^

Taken together, these findings suggest that the developmental impact of traditional screen exposure cannot be reduced to a simple measure of time. Instead, it reflects a dynamic interplay between quantity, content quality, and the social context of use.^4^, ^30^ This nuanced understanding provides an essential foundation for evaluating emerging forms of digital interaction, including those mediated by AI.

## AI‐BASED INTERACTION: A DIFFERENT PARADIGM

### What makes AI different?

AI introduces a fundamental shift in digital exposure by transforming passive or semi‐interactive media into dynamic, adaptive systems.^5^, ^31^ Unlike traditional screen‐based content, AI‐driven technologies can modify responses in real time based on user input and behavioral patterns, creating highly individualized interactions.^6^, ^32^, ^33^ This responsiveness enables AI systems to simulate aspects of contingent social interaction, distinguishing them from both passive media and conventional interactive applications.^7^, ^9^, ^34^

AI‐based interaction is not a uniform construct but includes technologies with different interactional and developmental characteristics. Although traditional media are primarily consumed, AI systems are increasingly engaged with as interactive or socially framed agents.^8^, ^9^, ^35^ These distinctions are important because AI systems differ substantially in their interactional characteristics and developmental implications. For conceptual clarity, Table 1 summarizes four major categories of AI interaction, conversational agents, adaptive learning systems, AI companions and social robots, and generative AI tools, together with their main features and potential developmental implications.

**TABLE 1:** Major categories of artificial intelligence (AI)‐based interaction and potential developmental implications

AI type	Main features	Potential developmental implications	References
Conversational agents	Simulated dialogue and contingent responses	Perceived reciprocity and pseudo‐social interaction	[^35^]
Adaptive learning systems	Personalized feedback and scaffolding	Enhanced cognitive engagement and skill acquisition	[^5^, ^6^, ^7^]
AI companions and social robots	Socially framed interaction with affective cues	Increased perceived social bonding and possible displacement of human interaction	[^37^, ^38^]
Generative AI and creative tools	Open‐ended content creation and problem‐solving	Flexible exploration but blurred boundaries between human and AI‐generated interaction	[^8^]

### Cognitive benefits

The adaptive and interactive nature of AI systems offers several potential advantages for cognitive development. Personalized feedback and real‐time responsiveness can enhance learning efficiency by aligning content with the child's developmental level and pace.^5^, ^6^ Studies on AI‐assisted instruction in secondary and higher education showed that AI‐driven platforms effectively support skill acquisition, particularly in domains such as language learning, numeracy, and problem‐solving.^7^, ^33^ One key mechanism underlying these benefits is scaffolding, the ability of AI systems to provide graduated support, guiding children through increasingly complex tasks.^7^ By adjusting difficulty and offering tailored prompts, AI can sustain engagement while promoting mastery. This may be particularly beneficial for children who require individualized learning approaches or who do not receive consistent instructional support in other environments.^33^, ^34^ Because this evidence derives from older learners rather than early‐childhood populations, the cognitive‐benefit claims above are generalized to early childhood by analogy rather than demonstrated directly in children under 5 years of age.

In addition, AI‐based interaction can encourage active cognitive engagement rather than passive consumption.^9^, ^36^ Tasks that involve decision‐making, hypothesis testing, and immediate feedback may strengthen executive functions, including working memory and cognitive flexibility.^36^ However, although these benefits are promising, they are often derived from structured or educational uses of AI, and their generalizability to unregulated or entertainment‐based contexts remains uncertain.^8^

### The illusion of social interaction

**FIGURE 1:**
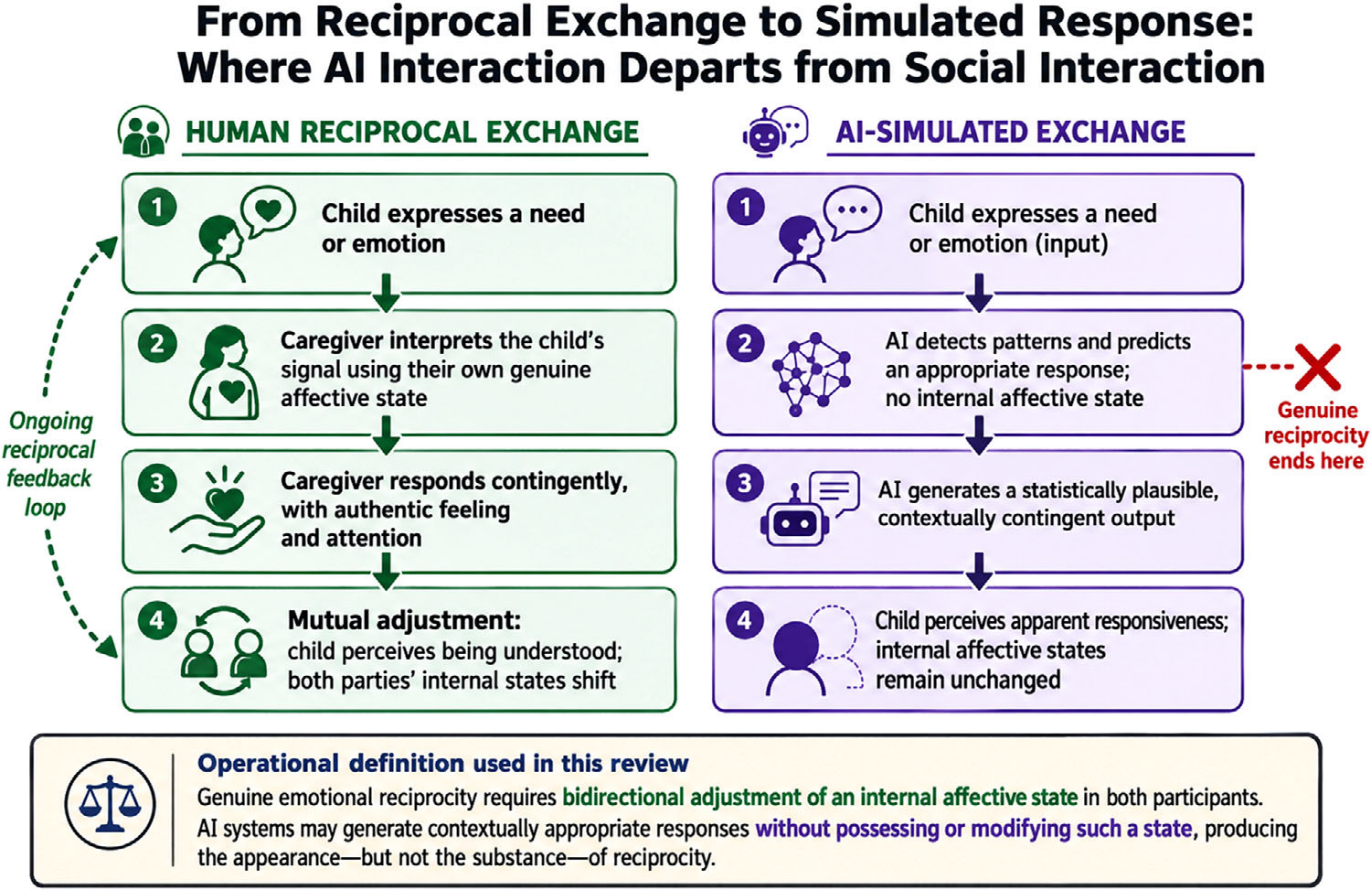
Mechanistic contrast between human reciprocal exchange and artificial intelligence (AI)‐simulated exchange. Genuine emotional reciprocity requires bidirectional adjustment of an internal affective state in both participants. AI systems can generate contingent, contextually appropriate responses without possessing or modifying such a state, producing the appearance, but not the substance, of reciprocity. This figure operationalizes the construct of genuine emotional reciprocity used throughout this review.

Despite its cognitive potential, AI‐based interaction raises important concerns regarding socio‐emotional development. A defining limitation of AI systems is the absence of genuine emotional reciprocity.^8^ Although AI can simulate conversational patterns and emotional responses, it does not possess authentic empathy, intentions, or social understanding. Consequently, interactions with AI may create an illusion of social engagement without providing the reciprocal dynamics necessary for developing real‐world social competence.^22^ This mechanistic gap between simulated and genuine reciprocity is illustrated in Figure 1. This may contribute to pseudo‐social bonding, in which children attribute relational qualities to AI systems and engage with them as social partners.^35^, ^37^ However, such interactions lack the unpredictability, emotional depth, and mutual responsiveness characteristic of human relationships.^22^ If AI‐based interaction displaces human engagement, opportunities to practice essential interpersonal skills, including interpreting social cues, negotiating perspectives, and emotional regulation, may be reduced.^37^ In addition, the predictability and controllability of AI responses may limit exposure to the social complexity required for resilience and adaptive social behavior.^7^, ^37^

At the same time, emerging evidence suggests that the developmental effects of AI are context dependent rather than uniformly harmful. Research on social robots and AI‐assisted interventions has shown potential benefits for supporting social engagement in children with autism spectrum conditions, particularly through structured and predictable interactions.^38^ Similarly, AI‐driven educational systems may enhance learning through scaffolding and individualized feedback.^7^ Children also appear to engage with AI in developmentally variable and context‐dependent ways rather than fully equating AI agents with human social partners.^35^ This pattern is reinforced by a more recent clinical systematic review.^39^ Collectively, this literature suggests that AI interaction shifts from a developmental substitute toward a developmental support under a definable set of conditions: When use is time‐bounded and goal‐directed rather than open‐ended, when a clinician, educator, or caregiver co‐directs the interaction toward a specific skill target, when the AI's behavior is predictable and low in ambiguity, and when the interaction is used to scaffold rather than replace opportunities for human contact. Outside these conditions, particularly in unstructured, companionship‐oriented, or unsupervised use, the substitution risks described above are more likely to dominate.

These findings suggest that AI‐based interaction may support cognitive engagement while also limiting opportunities for socio‐emotional development when it replaces human interaction.^8^, ^37^ Accordingly, the framework proposed in this review should be understood as a heuristic model highlighting general developmental tendencies rather than a deterministic trade‐off; the substitute‐versus‐support distinction outlined above defines the boundary conditions under which the model's general trade‐off is more or less likely to hold (Figure 1). A methodological caveat applies to several of the sources cited above: Uhls et al.^22^ reports data from a preteen sample at an outdoor camp, Festerling et al.^35^ studies primary‐school‐aged children, and Diehl et al.,^38^ draws on autism‐intervention research spanning a mixed age range that extends well beyond early childhood. These studies inform the present framework by analogy rather than by direct evidence in the target age group, and the socio‐emotional mechanisms they describe may operate differently, or with different intensity, in children under 5 years of age; this extrapolation should be borne in mind when interpreting the claims above.

### Generative AI companions and pseudo‐social relationships

The emergence of generative AI has introduced a new form of digital engagement that differs substantially from both traditional screen exposure and conventional interactive media. Large language model‐based systems, including conversational chatbots and AI companions, can sustain open‐ended dialogue, generate context‐sensitive responses, and create an impression of continuity across repeated interactions.^8^, ^34^ As a result, children may increasingly encounter AI not merely as a source of information but as a seemingly responsive conversational partner.

From a developmental perspective, these systems occupy a unique position within the digital environment because they blur the distinction between tool use and perceived social interaction. Research examining children's interactions with digital voice assistants has shown that children frequently attribute human‐like characteristics, intentions, and social qualities to conversational technologies.^35^ Generative AI systems may amplify this tendency because their responses are often more personalized, context‐aware, and linguistically sophisticated than those of earlier digital assistants.^8^ This phenomenon, best understood as a contemporary extension of the established parasocial‐relationship and attachment literature rather than as a wholly new one, raises concerns regarding the development of pseudo‐social relationships; children and adolescents have long been documented to form one‐sided emotional bonds with media figures, and a recent empirical study extends this tradition directly to conversational AI, finding that younger adolescents were more likely than older ones to personify an AI chatbot and to report positive emotional reactions to it.^40^ Although AI systems can simulate empathy, encouragement, and conversational reciprocity, they do not possess genuine emotional understanding, independent intentions, or mutual social responsibility. Consequently, interactions with AI companions may provide an experience of social engagement without exposing children to the interpersonal complexity required for developing empathy, perspective‐taking, conflict resolution, and emotional regulation.^23^, ^27^ The developmental significance of such interactions may be particularly relevant during early childhood, when socio‐emotional competencies are shaped primarily through reciprocal relationships with caregivers and peers.^4^, ^22^ Direct evidence of parasocial responses to AI within this specific age group remains limited, however, and the age‐graded pattern reported in older children and adolescents should be extrapolated to early childhood with caution.^40^

At the same time, potential risks should not be interpreted as evidence that AI‐mediated interaction is inherently harmful. Similar to other forms of digital engagement, developmental outcomes are likely to depend on context, content, parental involvement, and the extent to which AI use supplements or substitutes for human interaction.^3^, ^4^, ^28^ AI‐based systems may support learning, curiosity, and communication when integrated into developmentally appropriate environments.^5^, ^6^, ^7^, ^8^ However, excessive reliance on AI companions could alter opportunities for authentic social experiences and therefore warrants careful consideration in future pediatric research and policy development.^8^, ^31^

As generative AI technologies become increasingly accessible, understanding how children interpret, trust, and emotionally engage with AI companions should become a priority for developmental science. Future studies should distinguish AI‐mediated companionship from both traditional screen exposure and educational technology use, as these interactions may represent a developmentally distinct category of digital experience.

## CONCEPTUAL FRAMEWORK: A SPECTRUM MODEL OF DIGITAL EXPOSURE

The evolving landscape of digital technologies necessitates a shift from traditional time‐based metrics toward a more interaction‐focused framework of digital exposure.^4^, ^8^ Building on the limitations of conventional screen time paradigms, we propose a spectrum model that differentiates among passive screen exposure, interactive digital media, and AI‐based interaction. The model emphasizes that developmental outcomes are shaped not only by duration of use, but also by the quality and complexity of interaction.^13^, ^30^ The spectrum model presented here is built on the evidence reviewed above but has not been directly tested by it. In the sections that follow, we distinguish, where relevant, between findings supported directly by the literature and our conceptual interpretation of their interrelationships.

**FIGURE 2:**
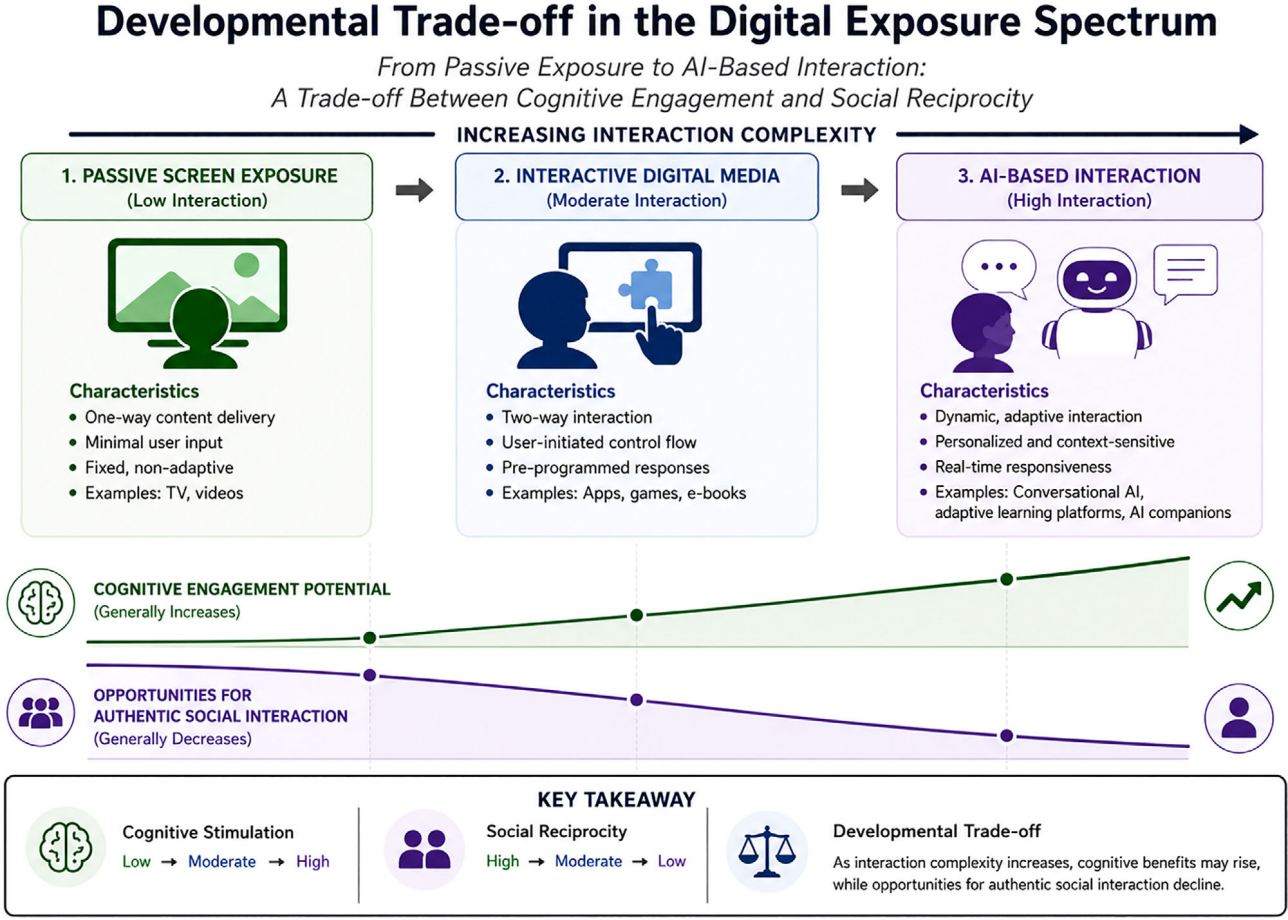
Developmental trade‐off in the digital exposure spectrum. Proposed conceptual model of the digital exposure spectrum. As interaction complexity increases from passive media to artificial intelligence (AI)‐based interaction, cognitive engagement generally increases, whereas opportunities for authentic social interaction tend to decrease. The framework illustrates a developmental trade‐off and is intended as a conceptual synthesis rather than an empirical effect‐size model.

As illustrated in Figure 2, these categories can be positioned along a continuum of interaction complexity, ranging from passive viewing to adaptive AI engagement. Passive screen exposure involves minimal user input and limited cognitive engagement, whereas interactive media introduce user control and responsiveness.^13^ AI‐based systems represent the highest level of interaction complexity through personalization, real‐time adaptation, and simulated dialogue.^5^, ^9^ Notably, increasing interaction complexity may differentially affect cognitive and socio‐emotional development. As summarized in Table 2, cognitive engagement does not necessarily increase in a linear fashion along this continuum; rather, it is likely to vary according to task design, developmental stage, content, and the degree of adult involvement, with AI‐based systems offering distinctive, though not uniformly superior, opportunities for personalized learning and problem‐solving support.^5^, ^7^ In contrast, opportunities for authentic social interaction may decrease when digital engagement displaces human relationships.^22^ Although AI systems can simulate social interaction, they do not provide genuine emotional reciprocity or the bidirectional relational dynamics required for socio‐emotional development, a distinction illustrated mechanistically in Figure 1.^8^, ^37^

**TABLE 2:** Developmental characteristics across types of digital exposure

Type of exposure	Typical age of predominant use	Observable marker	Cognitive and social engagement (illustrative)	Key developmental risk	Possible discussion point
**Passive screen exposure**	Infancy to preschool (from ∼6 months, increasing after age 2)	Prolonged noninteractive viewing (e.g., television, video content) without verbal exchange	Cognitive engagement: minimal, largely passive viewing. Social engagement: minimal—displaces caregiver interaction without offering reciprocity	Language delay, reduced sustained attention	Encourage co‐viewing; limit unstructured passive exposure; prioritize caregiver–child interaction
**Interactive digital media**	Toddlerhood through school age	User‐directed input (touch, choice‐making) without adaptive personalization	Cognitive engagement: greater than passive viewing, though dependent on task design. Social engagement: limited—turn‐taking structure without genuine reciprocity	Overuse, reduced real‐world play time	Set structured time limits; favor content requiring active problem‐solving; monitor for displacement of physical play
**AI‐based interaction**	Emerging in preschool and school age	Sustained conversational exchange with an AI system perceived by the child as responsive	Cognitive engagement: potentially substantial, but likely to vary according to task design, developmental stage, content, and the degree of adult involvement, and not necessarily equivalent to deeper learning. Social engagement: artificial/nonreciprocal—simulated responsiveness without genuine emotional exchange	Social substitution, pseudo‐social bonding, parasocial attachment	Assess purpose and context of use; encourage co‐engagement; screen for displacement of peer interaction

*Note*: This presents the authors’ conceptual synthesis of the developmental characteristics, observable markers, potential risks, and possible clinical responses associated with different forms of digital exposure. The qualitative impact descriptors are interpretive categories informed by the reviewed literature and should not be understood as validated categorical grades or measured effect sizes.

**FIGURE 3:**
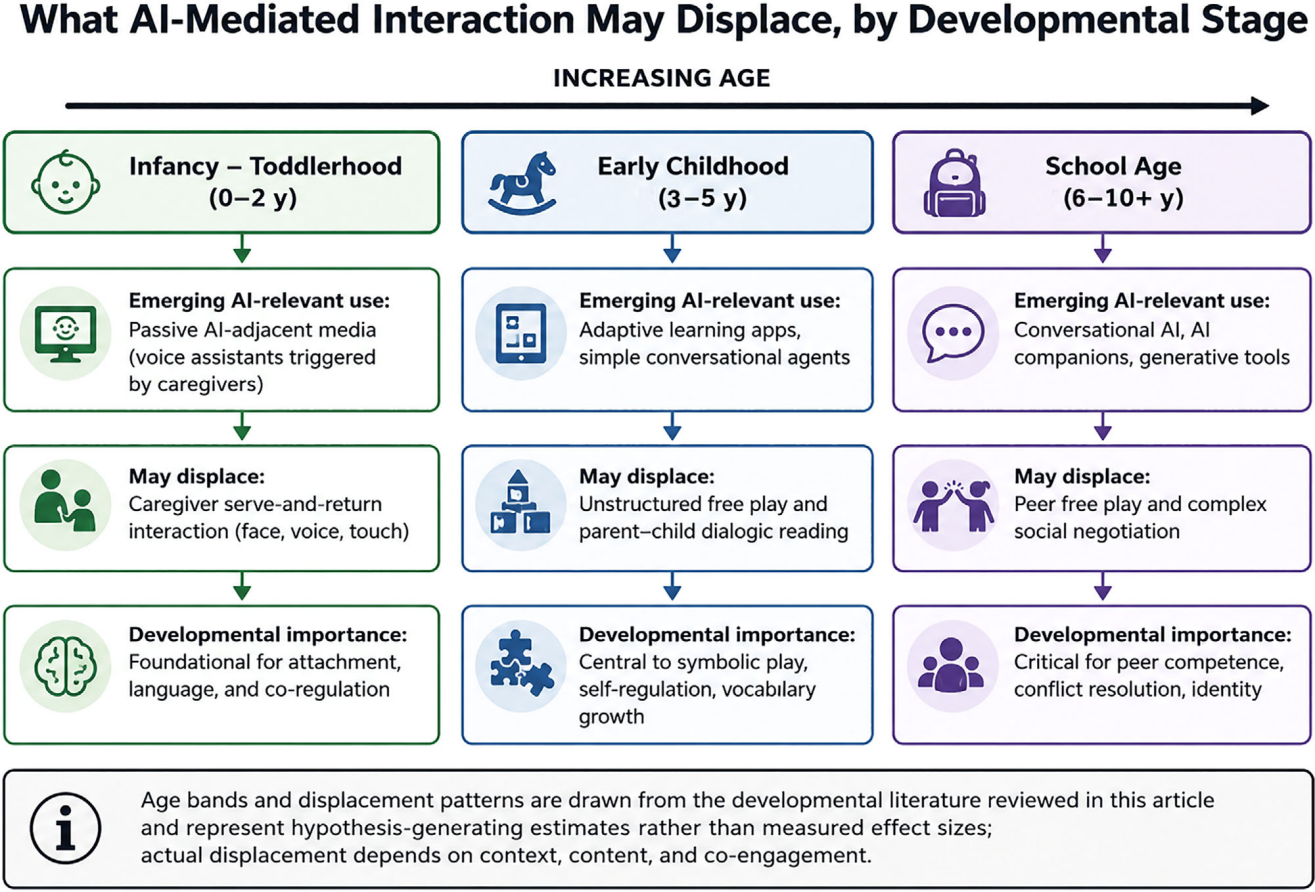
What AI‐mediated interaction may displace, by developmental stage. Illustrative mapping of the developmental activities that artificial intelligence (AI)‐mediated interaction may displace across childhood. As AI‐relevant use emerges at different developmental stages, it may replace activities of differing developmental importance. Age bands and displacement patterns represent hypothesis‐generating estimates derived from the developmental literature reviewed in this article rather than measured effect sizes.

The framework also highlights the importance of substitution effects, illustrated by developmental stage in Figure 3. We categorized children into three age groups: 0–2, 3–5, and 6–10 years, based on developmental rationale. Children begin to meaningfully comprehend child‐directed screen content at approximately 2 years of age, and distinguishable cognitive effects of media exposure become evident by the preschool years.^13^ The 6–10 group reflects the broader developmental shift that begins around school entry, made by a transition from caregiver‐ and peer‐mediated interaction toward more independent engagement with media. The developmental impact of digital exposure depends not only on the type of interaction, but also on what that interaction replaces.^23^, ^29^ For example, AI‐assisted learning may support cognitive development when supplementing traditional education, yet become detrimental if it substitutes for caregiver or peer interaction.^28^ As a first‐approximation operational marker, substitution can be estimated clinically by comparing when and how AI‐mediated interaction occurs relative to the human interaction it might displace: AI use that is solitary and unsupervised and that occurs during time otherwise available for caregiver or peer interaction (e.g., unstructured family time) constitutes a stronger substitution signal than AI use that is co‐engaged with a caregiver or that occurs alongside existing human interaction (e.g., structured educational time). Applying this heuristic, which corresponds to the co‐engagement and displacement domains in Table 3, should allow two clinicians reviewing the same history to arrive at a similar substitution classification, even though the underlying construct has not yet been validated psychometrically.

**TABLE 3:** Proposed domains for clinical assessment of artificial intelligence (AI)‐mediated interaction in early childhood

Assessment domain	Example question	Pattern warranting further discussion	Illustrative follow‐up consideration
Presence and type of AI interaction	Does the child interact with conversational AI systems (e.g., chatbots, virtual assistants, or AI companions)?	Regular, unsupervised use of an AI system that the child frames as a friend or confidant	Clarify context and frequency of use; introduce co‐engagement if none is currently present
Frequency and duration	How frequently, and for how long, does the child engage with these systems?	Daily or near‐daily use exceeding age‐appropriate digital exposure guidance	Compare against existing screen‐time benchmarks; consider discussing developmentally appropriate boundaries and alternative activities with the family
Purpose of use	What is the primary purpose of use—education, entertainment, emotional support, companionship, or information seeking?	Primary purpose is emotional support or companionship rather than education or entertainment	Explore why the child turns to the AI system for emotional needs; assess broader sources of social support
Co‐engagement context	Does AI use occur independently, or together with a parent, caregiver, or peer?	Exclusively solitary use with no adult involvement or mediation	Consider discussing opportunities for joint media use and parent–child interaction, where developmentally appropriate
Child's perception of the AI system	Does the child appear to treat the AI system as a tool, an information source, or a social partner?	Child attributes feelings, loyalty, or friendship to the AI system	Discuss developmentally appropriate framing of AI with the family; monitor for parasocial attachment
Displacement of face‐to‐face interaction	Has AI‐mediated interaction reduced opportunities for face‐to‐face interaction, free play, or peer engagement?	Clear reports that AI use is replacing peer play, family time, or outdoor activity	Assess for broader social withdrawal; consider referral if displacement is substantial or persistent

*Note*: The assessment domains and example questions above have not yet been formally validated; they are offered as a preliminary framework to guide history‐taking and will require validated psychometric development, including reliability and cross‐age comparability testing, before adoption as a formal screening tool.

Considered together, these dimensions provide a more precise framework for understanding the heterogeneous developmental effects of digital technologies and suggest that future research and clinical assessment should prioritize interaction characteristics rather than duration alone.^4^, ^8^ These conceptual distinctions also have important implications for pediatric assessment, caregiver practices, and future policy development.

## CLICIAL AND DEVELOPMENTAL IMPLICATIONS

### Implications for pediatric practice

Overall, the findings of this review suggest that current clinical approaches to assessing children's digital exposure may be insufficient in the context of rapidly evolving technologies. Pediatric evaluations have traditionally relied on quantifying “screen time” as a primary indicator of risk.^4^, ^30^ However, this metric does not adequately capture the qualitative differences between passive media consumption, interactive applications, and AI‐based engagement.^8^, ^27^

**FIGURE 4:**
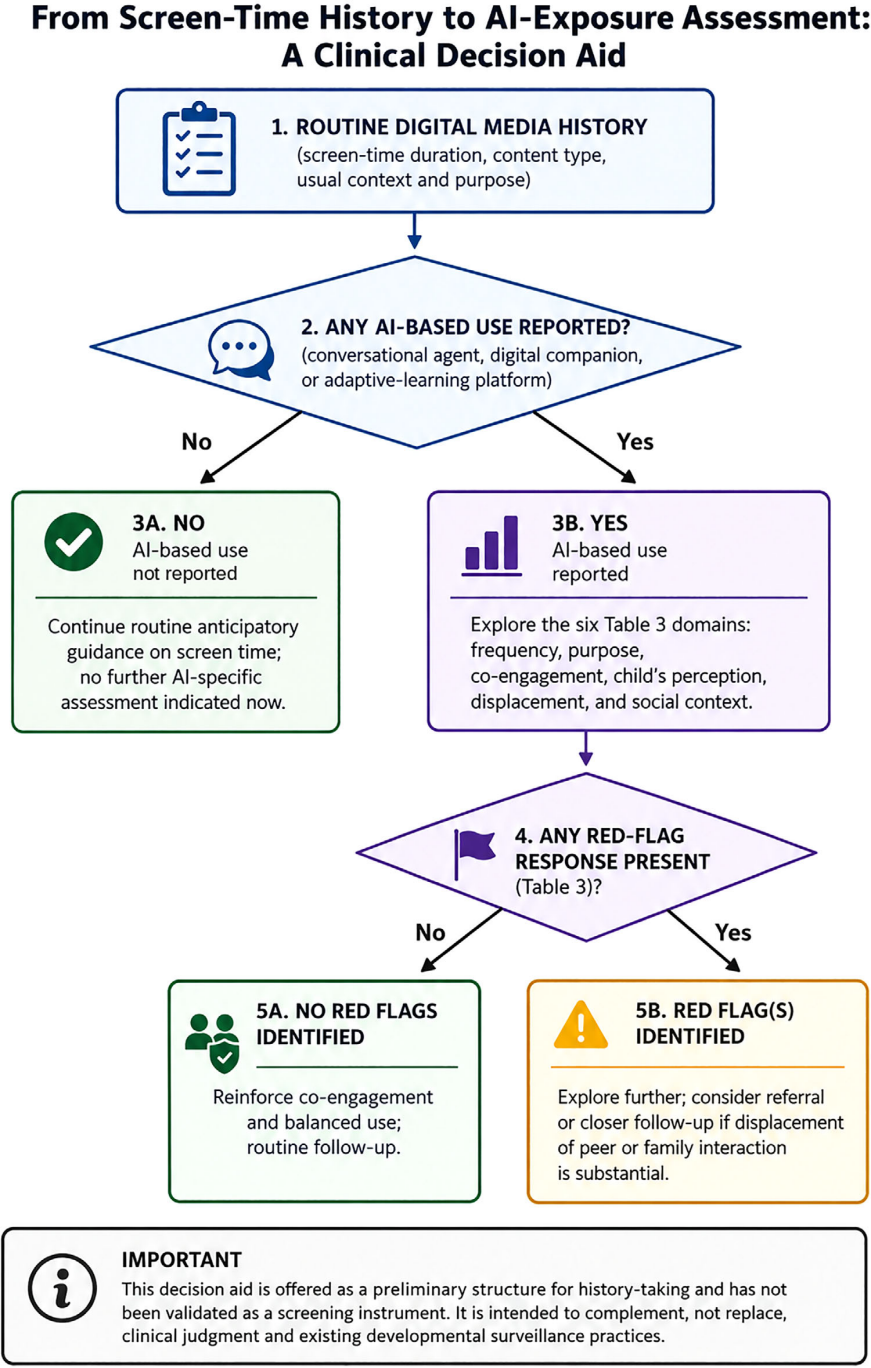
A clinical decision aid: from screen‐time history to artificial intelligence (AI)‐exposure assessment. Proposed framework for extending routine digital media history‐taking to AI‐mediated interaction, incorporating the assessment domains summarized in Table 3. This decision aid has not been validated as a screening instrument and is intended to complement, rather than replace, clinical judgment.

In light of the proposed spectrum model, which remains a conceptual synthesis rather than a validated clinical tool, pediatricians may consider expanding routine history‐taking to include not only the duration of digital exposure but also the type and nature of interaction.^4^, ^27^ Rather than remaining at the level of general advice, this expanded history‐taking can be operationalized directly using the six assessment domains and the accompanying decision aid presented in Table 3 and Figure 4, which give clinicians a concrete starting point rather than an abstract principle. Specifically, assessing “AI exposure,” including the use of conversational agents, adaptive learning tools, and AI‐driven companions, may provide a more accurate understanding of a child's developmental environment.^8^, ^34^ Questions regarding whether digital use is solitary or shared, structured or unstructured, and educational or entertainment‐based are equally important.^3^, ^29^ Furthermore, clinicians should be aware of the potential for AI systems to create an impression of meaningful interaction while lacking true social reciprocity. This distinction is particularly relevant when evaluating children with social or communication vulnerabilities, where reliance on AI‐based interaction could inadvertently reinforce social withdrawal.^37^

### Implications for parents and caregivers

For parents, the key implication is that not all digital engagement carries the same developmental weight. The concept of “quality over quantity” becomes increasingly important as AI technologies become more prevalent.^4^, ^29^ The framework proposed here would suggest that caregivers consider prioritizing how and with whom children engage with digital content.^28^ Co‐use, or shared media engagement between parent and child, remains a critical protective factor.^3^, ^23^ When caregivers actively participate, by discussing content, asking questions, and linking digital experiences to real‐world contexts, they can transform digital interaction into a socially enriched learning opportunity.^13^, ^29^ This is particularly important in the case of AI‐based tools, where the illusion of interaction may otherwise reduce the child's motivation to seek human engagement.^8^ Additionally, it is essential to avoid the use of AI systems as substitutes for human interaction. Although AI can support learning and provide stimulation, it should not replace caregiver involvement, peer interaction, or unstructured play, all of which are foundational for healthy socio‐emotional development.^22^, ^23^ Establishing boundaries around when and how AI tools are used can help maintain this balance.^30^

### Implications for policy and guidelines

At the policy level, current pediatric guidelines predominantly emphasize limits on screen time, often without differentiating between types of digital engagement.^4^, ^27^ Updated frameworks must incorporate interaction quality and technological characteristics.^8^, ^31^ This policy gap extends to data privacy and governance, which the current draft does not address: AI systems that interact with children by voice or text necessarily record and process sensitive behavioral and biometric data, and questions of what is collected, how long it is retained, and who can access it are a first‐order policy concern rather than a peripheral one. A recent compliance framework for secure health‐data exchange under Health Insurance Portability and Accountability Act (HIPAA) and General Data Protection Regulation (GDPR) illustrates the kind of governance structure, encompassing data minimization, consent, and auditable access control, that child‐facing AI systems would need to meet before their routine use in pediatric contexts could be considered privacy‐protective.^41^

Future guidelines should involve defining age‐appropriate uses of AI, recommending co‐use practices, and emphasizing the importance of maintaining human‐centered developmental experiences.^31^, ^34^ Policymakers should consider the rapid integration of AI into educational and domestic environments, ensuring that these technologies are implemented in ways that support, rather than undermine, developmental outcomes.^5^, ^31^ This includes promoting transparency in AI design, encouraging the development of child‐centered ethical standards, and supporting research that evaluates long‐term effects of AI exposure.^8^, ^42^

## DISCUSSION

### Synthesis and contribution to the literature

The rapid emergence of AI‐mediated interaction challenges conventional assumptions underlying pediatric screen time frameworks. The present review extends existing literature on digital media and child development by differentiating AI‐based interaction from traditional screen exposure. Although prior research has linked excessive screen time to adverse cognitive and socio‐emotional outcomes,^1^, ^2^, ^3^, ^13^ most frameworks continue to conceptualize digital exposure primarily in terms of duration.^4^, ^30^ The findings synthesized here support growing concerns that such approaches may not adequately capture the complexity of contemporary AI‐mediated environments.^8^, ^27^

This perspective aligns with recent calls to reconsider conventional “screen time” frameworks in the context of AI use. Nagata and Tsuchiya^8^ highlighted the need for AI‐specific epidemiological measures and longitudinal surveillance, particularly for generative AI use. Building on this work, the present review adopts a developmental perspective centered on early childhood, broadens the scope to multiple forms of AI interaction, and proposes the digital exposure spectrum as a conceptual framework linking interaction complexity with cognitive engagement and social reciprocity. The proposed framework contributes to the literature by distinguishing forms of digital interaction qualitatively rather than solely quantitatively. By positioning AI‐based interaction as high in cognitive engagement but limited in authentic reciprocity, the model helps reconcile findings showing both educational benefits of interactive technologies^7^, ^33^ and their potential to displace human interaction.^22^, ^23^ This duality may be particularly relevant in early childhood, where developmental processes are highly dependent on both cognitive stimulation and interpersonal experience.^18^, ^22^

### Limitations

Several limitations should be acknowledged. Direct empirical evidence regarding AI‐based interaction in early childhood remains limited,^8^, ^34^ and much of the existing literature focuses on educational or experimental contexts.^5^, ^7^ In addition, the heterogeneity of AI systems, ranging from simple conversational agents to highly adaptive platforms, complicates generalization across technologies.^9^, ^31^ Consequently, some arguments presented here remain conceptual and hypothesis‐generating rather than empirically definitive. Two further limitations should be acknowledged. First, as a narrative rather than systematic review, this synthesis carries an inherent degree of selection latitude: The authors identified and prioritized sources based on topical relevance and conceptual fit rather than a pre‐registered, exhaustive search protocol, and the review leans on a small number of anchor sources for several of its central claims; the resulting synthesis should accordingly be read as reflecting the authors’ framing of the available literature rather than an exhaustive account of it. Second, this review does not address equity and access: opportunities for adaptive, personalized AI use, and for the adult co‐engagement that appears to moderate its developmental impact, are likely to differ by household resources, digital literacy, and access to devices and connectivity, so the cognitive benefits and social risks discussed here may not be distributed evenly across socioeconomic groups.

### Future research directions

Future research should prioritize longitudinal and naturalistic studies examining the developmental effects of AI exposure across different contexts of use.^8^, ^30^ Greater differentiation between educational, social, and entertainment‐oriented AI systems will also be important, particularly with respect to interactivity, co‐engagement, and developmental timing.^34^, ^42^ Adjacent health and performance fields have begun to apply individualized computational modeling, such as digital‐twin frameworks that integrate longitudinal, person‐specific data to predict how a given individual's outcomes evolve under varying conditions.^43^ An analogous approach, adapted to relevant developmental and behavioral indicators, could give this future‐research agenda a concrete methodological direction: Modeling how a given child's cognitive and social trajectories evolve under differing patterns of AI use, rather than relying solely on group‐level associations.

The increasing integration of AI into children's daily environments suggests that pediatric assessment may need to move beyond traditional screen‐time measures. In addition to quantifying overall digital exposure, clinicians may benefit from exploring the nature and context of AI‐mediated interactions, structured here across six assessment domains (Table 3). As AI technologies become increasingly embedded in childhood environments, the development of standardized measures of AI‐mediated interaction may represent an important future direction for pediatric surveillance and developmental screening.^8^, ^27^

The conceptual framework proposed here represents an initial step toward understanding how AI‐mediated interaction may shape developmental trajectories, distinguishing it from passive screen exposure through its emphasis on interaction quality, co‐engagement, and substitution effects.

## CONCLUSION

AI represents a qualitatively different form of digital exposure that may not be fully captured by traditional screen time frameworks. Unlike passive media, AI‐based systems can provide adaptive and personalized interaction, which may increase cognitive engagement. At the same time, and consistent with the operational distinction outlined in Figure 1, AI‐mediated interaction does not meet the criteria for genuine emotional reciprocity, despite simulating aspects of social communication. Consequently, although AI systems may support learning and engagement, they may also constrain socio‐emotional development if they displace meaningful human interaction.

Future research, clinical assessment, and policy frameworks should therefore move beyond duration‐based measures of screen exposure and place greater emphasis on the quality and context of digital interaction. As AI technologies become increasingly integrated into children's everyday environments, AI exposure may warrant consideration as a distinct developmental exposure category in future pediatric research. Such an approach may facilitate the development of more precise assessment tools, age‐appropriate guidelines, and evidence‐based policies that better reflect the developmental realities of AI‐mediated childhood experiences.

## CONFLICT OF INTEREST

The authors declare no conflicts of interest.
